# Novel Synthesis of Kanamycin Conjugated Gold Nanoparticles with Potent Antibacterial Activity

**DOI:** 10.3389/fmicb.2016.00607

**Published:** 2016-05-02

**Authors:** Jason N. Payne, Hitesh K. Waghwani, Michael G. Connor, William Hamilton, Sarah Tockstein, Harsh Moolani, Fenil Chavda, Vivek Badwaik, Matthew B. Lawrenz, Rajalingam Dakshinamurthy

**Affiliations:** ^1^Department of Chemistry, Western Kentucky University, Bowling GreenKY, USA; ^2^Center for Predictive Medicine for Biodefense and Emerging Infectious Diseases and the Department of Microbiology and Immunology, University of Louisville School of Medicine, LouisvilleKY, USA; ^3^Department of Chemistry, Austin Peay State University, ClarksvilleTN, USA

**Keywords:** gold nanoparticles, kanamycin, antibiotic resistance, antibacterial activity, characterization

## Abstract

With a sharp increase in the cases of multi-drug resistant (MDR) bacteria all over the world, there is a huge demand to develop a new generation of antibiotic agents to fight them. As an alternative to the traditional drug discovery route, we have designed an effective antibacterial agent by modifying an existing commercial antibiotic, kanamycin, conjugated on the surface of gold nanoparticles (AuNPs). In this study, we report a single-step synthesis of kanamycin-capped AuNPs (Kan-AuNPs) utilizing the combined reducing and capping properties of kanamycin. While Kan-AuNPs have increased toxicity to a primate cell line (Vero 76), antibacterial assays showed dose-dependent broad spectrum activity of Kan-AuNPs against both Gram-positive and Gram-negative bacteria, including Kanamycin resistant bacteria. Further, a significant reduction in the minimum inhibitory concentration (MIC) of Kan-AuNPs was observed when compared to free kanamycin against all the bacterial strains tested. Mechanistic studies using transmission electron microscopy and fluorescence microscopy indicated that at least part of Kan-AuNPs increased efficacy may be through disrupting the bacterial envelope, resulting in the leakage of cytoplasmic content and the death of bacterial cells. Results of this study provide critical information about a novel method for the development of antibiotic capped AuNPs as potent next-generation antibacterial agents.

## Introduction

Since the discovery of the first antibiotic, Penicillin, in 1928 (National Historic Chemical Landmarks program, 1999) the process of developing mechanisms of resistance against various synthetic antibiotics has been initiated in bacteria. This is evident from the presence of various resistant bacterial strains in the early 1930s and 1940s (Clatworthy et al., 2007; “News Feature: A Call to Arms”, 2007). Moreover, widespread and irrational use of antibiotics across the globe has led to the emergence of antibiotic resistant strains of bacteria. A number of bacterial strains are even resistant to multiple antibiotics, known as multi-drug resistant (MDR) bacterial strains (Jugheli et al., 2009; Arias and Murray, 2012; Bennadi, 2013; Laxminarayan et al., 2013; Sutradhar et al., 2014; WHO | Antimicrobial Resistance, 2014; “Antibiotic Resistance: An Ecological Perspective on an Old Problem”, 2015; “Antibiotic Resistance Threats in the United States, 2013”, 2015; World Health Organization [WHO], 2015). Currently the rate at which bacteria are developing resistance to existing antibiotics is faster than the development of newer antibiotics (“World Economic Forum – Global Risks 2013 Eighth Edition”, 2014). Several strategies are being employed to develop novel antibacterial agents or to potentiate the activity of currently existing/commercially successful antibiotics (Tillotson and Theriault, 2013). One of the most widely researched approaches involves the use of metallic and metal-oxide nanoparticles (1–100 nm), such as gold (Au), silver (Ag), and zinc oxide (ZnO) to enhance bactericidal activity (Seil and Webster, 2012; Chernousova and Epple, 2013).

Among various inorganic nanomaterials, gold nanoparticles (AuNPs) have gained immense attention for the design and development of innovative biomedical tools (Bao et al., 2013; Drbohlavova et al., 2013; Jiang et al., 2013; Khan et al., 2013; Khlebtsov et al., 2013; Kumar et al., 2013; Zhao and Jiang, 2013; Zhang et al., 2013; Zhou et al., 2013; Liao et al., 2014; Shah et al., 2014). AuNPs have a number of inherent features, such as biosafety (Finkelstein et al., 1976; Stanier, 2014), ease of functionalization (Tiwari et al., 2011), facile synthesis (Akhtar et al., 2013; Shah et al., 2014; Shedbalkar et al., 2014), large surface/volume ratio allowing the release of high drug payload at infected sites (Voliani et al., 2012), multiple-targets of bactericidal action (Tillotson and Theriault, 2013), and an ability to penetrate biological membranes (Voliani et al., 2012; Tillotson and Theriault, 2013), which make them strong candidates for the development of novel antibacterial agents. Despite the fact that AuNPs are strong candidates for antibacterial agents, AuNPs have been much more widely utilized as nanoparticles for cancer therapy (Ahmad et al., 2013; Drbohlavova et al., 2013; Almeida et al., 2014), while less extensive efforts have been made in the field of antibacterial agents (Zhao and Jiang, 2013). Complicating the utilization of AuNPs for antibacterial treatment is the fact that common methods of synthesis involve multiple steps, use toxic organic chemicals, and occur under harsh reaction conditions, all of which could have detrimental effects on biological systems (Burygin et al., 2009; Zhao et al., 2010; Bhattacharya et al., 2012; Brown et al., 2012; Zhou et al., 2012; Lima et al., 2013).

Kanamycin is an aminoglycoside antibiotic with broad spectrum activity that inhibits bacterial protein synthesis (Umezawa, 1958). High water-solubility (50 mg/mL) and absence of delayed toxicity are some of the additional advantages of kanamycin over other actinomycete-derived antibiotics (Xie et al., 2011). Here, we attempt to potentiate the antibacterial activity of kanamycin by designing a unique formulation of kanamycin capped gold nanoparticles (Kan-AuNPs) using a simple, kanamycin-mediated, bio-friendly synthesis that we have developed (Dakshinamurthy and Sahi, 2012; Pender et al., 2013). To this end we established and fine-tuned the synthesis protocol by determining the optimum concentrations of nucleating agent [potassium gold (III) chloride (KAuCl_4_)], and reducing agent (kanamycin sulfate) to yield stable, monodispersed Kan-AuNPs. The resultant Kan-AuNPs were morphologically characterized by various analytical and spectroscopic techniques. The Kan-AuNPs were then evaluated for their antibacterial activity against multiple bacterial strains. Kan-AuNPs showed a significant improvement in efficacy against both susceptible and resistant Gram-positive and Gram-negative bacterial strains as compared to pure kanamycin. Finally, electron microscopy revealed that Kan-AuNPs localized on the entire surface of the bacterial cell envelope eventually resulting in bacterial lysis. The results demonstrate a successful development of a highly efficient antibiotic-AuNP against several infectious bacterial strains suggesting a novel strategy to combat MDR bacteria.

## Materials and Methods

### Materials

Analytical grade kanamycin sulfate (Calbiochem, EMD Chemicals), potassium gold(III) chloride (Sigma Aldrich), sodium citrate dihydrate (Sigma Aldrich) Luria-Bertani (LB) broth, (Novagen, EMD Biosciences), Tryptic soy (TS) broth (Teknova), Lennox LB agar, [Beckton, Dickson (BD) and Co.], TS agar (BD and Co.), XTT (Biotrium), Menadione (Spectrum chemicals), and propidium iodide (Calbiochem) were purchased and used as required. All laboratory glassware was thoroughly cleaned and rinsed followed by steam sterilization (121°C, 45 min) before use. The bacterial organisms *Staphylococcus epidermidis* (ATCC #12228), *Streptococcus bovis* (ATCC # 9809), *Enterobacter aerogenes* (ATCC # 13048), *Pseudomonas aeruginosa* PA01, *P. aeruginosa* UNC-D (Lawrenz et al., 2015), *Yersinia pestis* CO92 Lux_P_*_tolC_* pCD1^(-)^ (Sun et al., 2012), and *Y. pestis* CO92::Km Lux_P_*_tolC_* pCD1^(-)^ (*Y. pestis* CO92 Lux_P_*_tolC_* pCD1^(-)^ with unresolved Kan resistance cassette) were cultured by standard procedures.

### Biofriendly Synthesis of Kanamycin-Gold Nanoparticles (Kan-AuNPs)

1.72 mM kanamycin sulfate dissolved in an M9 minimal media buffer (pH 7.2 ± 0.2) was heated to 80°C and aqueous KAuCl_4_ was added to achieve a final concentration of 0.79 mM KAuCL_4_. The mixture was incubated for 5 min and then allowed to cool to room temperature. A colorimetric shift from colorless to pink indicated the formation of Kan-AuNPs. Unreacted kanamycin (Kan) was removed by at least five cycles of washing and centrifugation (15,000 rpm for 20 min). Following the final wash, Kan-AuNPs were pelleted, freeze-dried, and stored at room temperature. Freeze dried Kan-AuNPs were re-suspended in autoclaved nanopure water for subsequent analysis.

### Characterization of Antibiotic Coated Nanoparticles

The size and shape of Kan-AuNPs was determined using a JEOL JEM 1400 Plus transmission electron microscope (TEM) with 120 kV accelerating voltage. For analysis, 5 μL of Kan-AuNPs suspension was placed on a 400-mesh formvar-coated copper grid and allowed to air-dry. Images of the selected regions were taken using the built-in AMT XR-81M-B camera which were later developed and processed via Capture Engine Software AMT Version 602.600.52. The UV-Vis spectrum of probe sonicated samples of Kan-AuNPs was determined using a Hitachi U-3900 spectrophotometer at a resolution of 0.5 nm to observe the peaks generated by the characteristic optical properties of AuNPs known as surface plasmon resonance (SPR). Diameter and average size distribution of the particles was determined using a Zetasizer Nano S (Malvern Instruments Ltd.) dynamic light scattering analyzer at 25°C with a scattering angle of 90°. One mililiter of 2 mg mL^-1^ Kan-AuNPs suspension was probe sonicated and the average of the three measurements involving 13 runs each were performed. Presence of Kan on the surface of the AuNPs was confirmed from the surface elemental analysis using a JEOL-JSM-6510 LV scanning electron microscope (SEM) with IXRF system. Fifty microliter of 1 mg mL^-1^ sonicated sample of Kan-AuNPs suspension was evenly spread on a cleaned silicon wafer and dried under vacuum. SEM images of substrate were obtained at 20 kV accelerating voltage and 5kX magnification followed by surface elemental analysis using energy dispersive spectroscopy (EDS). The presence and percentage of Kan (w/w) was confirmed using thermo-gravimetric analysis (TGA). Approximately 5 mg of lyophilized Kan-AuNPs and pure Kan powder was heated individually in a platinum pan over a temperature range of 25°C to 850°C at a heating rate of 10°C min^-1^ in the presence of nitrogen (N_2_) gas using a TA Q5000 instrument. The N_2_ gas was changed to air after 650°C to allow complete oxidation. A thermogram showing the gradual weight loss of ligand upon heating of Kan-AuNPs was plotted and compared to pure Kan. The electrostatic charge and stability of the nanoparticles were determined using a Zetasizer Nano S. A 1 mg mL^-1^ Kan-AuNP suspension was prepared by dissolving the sample in a 10 mM NaCl solution followed by filtration through a 0.1 μm pore size filter. The pH of the solution was maintained at a pH of 7.2 ± 0.2 throughout the analysis. The analysis was performed at 25 ± 0.3°C, with an equilibration time of 2 min, and the applied voltage was 100 V. Analysis was performed in triplicate to verify the results (Nanotechnology Characterization Laboratory, 2009).

### Synthesis and Characterization of Citrate-AuNPs

For synthesis of citrate conjugated gold nanoparticles the Turkevich method was employed (Turkevich et al., 1951). Briefly, 1 mM Aurochloric acid was preheated to 100°C with a 1% solution of sodium citrate dihydrate (Kimling et al., 2006). A colorimetric shift from colorless to dark red was indicative of the formation of Citrate-AuNPs. The reaction mixture was allowed to cool to room temperature and the resulting solution was titrated to a pH of 7.2 ± 0.2. Unbound reactants were removed by washing with autoclaved nanopure water and centrifugation (15,000 rpm for 20 min) five times. After the final wash, Citrate-AuNPs were pelleted, lyophilized, and stored at room temperature until resuspended for use. Citrate-AuNPs were characterized by TEM and zeta potential as described above for Kan-AuNPs.

### Evaluation of Kan-AuNPs for Antibacterial Activity

Twofold serial dilutions of the Kan, Kan-AuNPs, or Citrate-AuNPs were made in a 96 well microtiter plate as previously described (National Committee for Clinical Laboratory Standards, 1999; Wiegand et al., 2008). Bacteria were grown overnight and 3 × 10^5^ colony forming units (CFU) were added to each well. One well at each antibiotic concentration was inoculated with medium alone as a sterility control. For the positive growth control, the same volume of antibiotic was replaced with equivalent amount of nutrient medium (National Committee for Clinical Laboratory Standards, 1999). The plates were statically incubated at 37°C for 24 h. The minimum inhibitory concentrations (MIC) were determined to be the antibiotic concentration that completely inhibited bacterial growth with respect to the positive control (National Committee for Clinical Laboratory Standards, 1999). Each experiment was performed in triplicate to verify the validity of the results. In order to directly compare concentrations of antibiotic required to inhibit growth, the MIC of Kan-AuNPs represents the concentration of the Kan present in each well (as calculated using the organic and inorganic compositional values determined through TGA). When comparing Citrate-AuNPs to Kan-AuNPs, the MIC represents the concentration of the gold present in each well.

### Cellular Cytotoxicity of Kan-AuNPs

To determine host cell cytotoxicity, 2 × 10^4^ Vero 76 cells in DMEM + 10% FBS were added to wells of a white CellStar 96 well plate (Greiner Bio-One). 24 h later, the medium was replaced with twofold serial dilutions of Kan-AuNP in DMEM + 10% FBS (final concentrations from 0.002 to 5.0 mg ml^-1^) or Kan (final concentrations from 0.04 to 160 mg ml^-1^) and returned to the incubator. After 24 h, 10 μl of AlamarBlue (Invitrogen) was added and incubated for 2 h. AlamarBlue fluorescence was determined using a Synergy H1 plate reader (BioTek) and compared to untreated Vero 76 cells. The cytotoxic concentration was calculated using the log(inhibitor) vs. response equation in Graph Pad Prism 5.

### Evaluation of Interactions of Kan-AuNPs with Bacteria

To visualize morphological changes in bacteria after inoculation with Kan-AuNPs, cross-sections of bacteria were prepared with the aid of ultra-microtome and observed under a TEM (Badwaik et al., 2011, 2013). One mililiter of bacterial culture was incubated at 37°C, 150 rpm, for 12 h in the presence of the MIC of Kan-AuNPs. The sample was then centrifuged (at 4,000 rpm, for 3 min) and the pellet was re-suspended in 1 mL of primary fixing solvent (16% w/v paraformaldehyde and 10% w/v glutaraldehyde in 50 mM sodium cacodylate buffer [pH ∼7.4]) and incubated for 2 h. After incubation, bacteria were centrifuged and washed twice with cacodylate buffer. Bacteria were then re-suspended in 1 mL of 1% osmium tetroxide solution (OsO_4_), and further incubated for 1 h at 25°C for post-fixation. Post-fixed samples were treated with graded ethanol concentrations (25, 50, 75, 95, and 100%) after washing with nanopure water. The dehydrated samples were centrifuged and the pellets were infiltrated with graded Spur’s epoxy resin (33, 66, 95, and 100 %) for an hour and left overnight in 100% resin. The samples were then centrifuged in BEEM^®^ capsules, which were solidified by heating at 70°C for 18 h. Using glass knives for RMC MT-X ultra-microtome, ultra-thin sections of sample were cut and stained with 2% aqueous uranyl acetate for 15 min and Reynold’s lead citrate for 3 min. The samples were then imaged with a JEOL-100CX TEM.

Bacterial membrane permeability was evaluated by propidium iodide (PI) exclusion (Zhao et al., 2010). Bacteria were incubated in the presence of the MIC of Kan-AuNPs for ∼12 h, washed with phosphate buffer saline (PBS) and centrifuged (6000 rpm, 3 min) thrice. Washed bacteria were incubated with 100 μM PI for 30 min in the dark. After incubation, unbound PI was removed by washing the sample with PBS. Ten microliter of the resulting suspension was placed on a glass slide and covered with a cover slip. The sample was viewed using a Leica fluorescence microscope. Bacteria without Kan-AuNPs inoculation served as the negative control. The number of permeable cells (showing red fluorescence) was calculated as an average from three individual measurements.

## Results and Discussion

### Synthesis and Characterization of Kan-AuNPs

One of the vital steps in the preparation of AuNPs is the addition of chemical agents to reduce the gold ions (Au^3+^/Au^2+^) to neutral gold atoms, which results in aggregation upon reaching the saturation limit (Shah et al., 2014). In traditional synthesis protocols a secondary capping agent is then added to stabilize and restrict the size of gold aggregates to the nanometer size range (Shah et al., 2014). However, using such methods requires multiple steps to purify the product of unwanted components, which results in the overall process being expensive and labor intensive with limited scalability. Furthermore, due to concerns regarding the biological compatibility of the chemical agents used in the synthesis process, there has been a recent emphasis on finding biologically friendly methods for synthesizing AuNPs (Shah et al., 2014). In this context we developed a simple, single-step method for synthesizing antibiotic functionalized AuNPs. Kan has electron rich hydroxyl and amine functional groups which can serve as both a reducing and capping agent. The optimum concentration of Kan and gold required for AuNP synthesis was determined by assessing varying concentrations of both Kan and KAuCl_4_ (data not shown). Based on these analyses it was determined that 1.72 mM of Kan and 0.79 mM of KAuCl_4_, when incubated in aqueous buffer (pH ∼7.2 ± 0.2 heated to 80°C), for 5 min, followed by cooling to 37°C, formed AuNP of uniform size and dispersion. This synthesis protocol resulted in the optimal yield based on the size and morphological character of Kan-AuNPs (see below).

**FIGURE 1:**
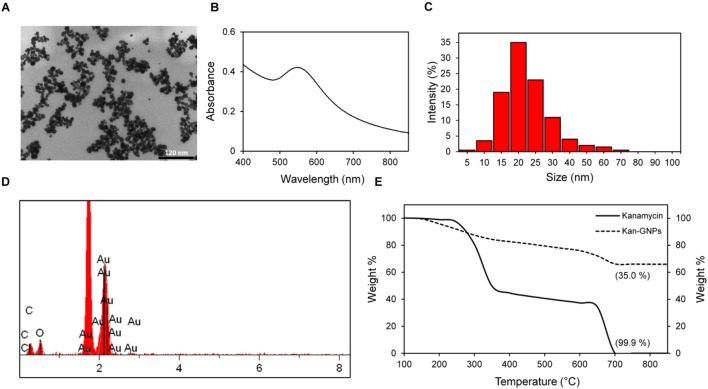
**Illustration of the morphological characterization of synthesized Kan-AuNPs.** **(A)** TEM micrograph showing the formation of well-dispersed spherical Kan-AuNPs in the size range of 20 ± 5 nm. **(B)** UV-Vis spectra of Kan-AuNPs showing a strong absorption peak at 546 nm which is characteristic of spherical AuNPs. **(C)** Plot showing average particle size distribution of Kan-AuNP suspension obtained using DLS **(D)** Energy dispersive spectroscopy (EDS) spectra of Kan-AuNPs showing the presence of an elemental peak for carbon (C) and gold (Au) at 0.2 and 2.1 keV respectively. Figure in the inset shows SEM image of spin coated sample of Kan-AuNPs on silicon chip obtained at an accelerating voltage of 20 keV with a magnification of 5 kX. **(E)** A comparison of thermo-gravimetric (TGA) analysis showing loss of organic material for kanamycin (**—**) and Kan-AuNPs (**- - -**) respectively. The samples were heated from room temperature to 650°C at a rate of 10°C min^-1^ under nitrogen flow followed by heating till 850°C under air.

Qualitative analysis of aggregation behavior of nanoparticles and morphological characteristics were studied using TEM at 120 kV. Micrographs showed the particles to be nearly spherical shaped and monodispersed with an average diameter of 20 ± 5 nm (**Figure 1A**). A UV-Vis spectrum of Kan-AuNPs recorded in the visible region (400–850 nm) showed a peak absorption (λ_max_) value at 546 nm (**Figure 1B**) which further supports the size of AuNP obtained from TEM (Haiss et al., 2007; Amendola and Meneghetti, 2009). Further, dynamic light scattering (DLS) analysis of Kan-AuNPs showed a sharp peak with an average size distribution of 20 ± 5 nm (**Figure 1C**) which is in agreement with TEM and UV-Vis data.

SEM-EDS analysis further defined the surface elemental composition of Kan-AuNPs, where dense portions of the Kan-AuNPs sample were selected and magnified during acquisition. The electron beam was focused on the selected region to obtain the localized elemental composition using IXRF software and the final elemental composition was averaged from three different regions of analysis. Results of the EDS spectral analysis revealed the presence of elemental peaks for carbon (C) and gold (Au) at ∼0.2 keV and ∼2.1 keV, respectively (**Figure 1D**). The percent composition of carbon and gold was found to be 16.65 and 66.55% respectively. Presence of a gold peak ensured the formation of AuNPs whereas the carbon peak confirmed the presence of the organic ligand (Kan) on the surface of AuNPs. Thermo-gravimetric analysis (TGA) of Kan-AuNPs was used to quantify the amount of organic ligand (Kan) bound to the surface of AuNPs. This measurement is essential to define the mass of Kan present on the surface of Kan-AuNPs, and thus, allowing for direct comparison between Kan and Kan-AuNPs in subsequent efficacy trials. The weight percentage is also critical in calculating the actual dose of drug for *in vivo* studies. From the thermogram, we observed a 35% loss in the total mass of heated Kan-AuNPs (**Figure 1E**). TGA of pure Kan was also performed for reference as shown in **Figure 1E.** The similar decomposition rates of Kan and the organic conjugate on the AuNPs indicate that Kan is conjugated to the AuNPs and comprises about 350 mg per 1.000 g of Kan-AuNP. Furthermore, the surface zeta potential of the Kan-AuNPs was determine to be +18.7 mV, indicating that the nanoparticles will readily interact with negatively charged bacterial membranes (Ivanov et al., 2009).

### Kanamycin Conjugation to AuNPs Improves Efficacy of the Antibiotic against Both Gram-Negative and Gram-Positive Bacteria

To determine whether AuNP conjugation increased the efficacy of Kan, we measured the MICs of Kan-AuNPs for a panel of bacterial species with varying degrees of resistance to Kan. For our initial analysis we chose several bacteria available from ATCC commonly used in the laboratory as controls for MIC assays (*S. bovis*, *S. epidermidis*, *E. aerogenes*) and *P. aeruginosa* PA01 and *Y. pestis* CO92. For each strain, Kan and Kan-AuNP MICs were determined using the broth culture microdilution method. As shown in **Table 1**, *S. bovis*, *S. epidermidis*, *E. aerogenes* and *P. aeruginosa* PA01 were relatively resistant to Kan, with MICs ranging from 50 to 512 μg/ml. Conversely, *Y. pestis* CO92 was sensitive to Kan with an MIC of 4.8 μg/ml. However, regardless of Kan sensitivity, when incubated with Kan-AuNPs, the Kan MIC dramatically decreased for all strains, with MICs <10 μg/ml Kan when conjugated to the AuNPs (**Table 1**). Furthermore, these inhibitory concentrations were also bactericidal, as determined by subculture onto non-selective agar (data not shown). Next we examined whether AuNP conjugation improved the efficacy of Kan against a MDR *P. aeruginosa* (UNC-D) and a genetically engineered *Y. pestis* Kan resistant strain (*Y. pestis*::Km). These strains represent potential clinical isolates (UNC-D) or manipulated biodefense species (*Y. pestis*::Km) that could be encountered in the hospital. The MIC of Kan against these strains was significantly higher than both *P. aeruginosa* PA01 and *Y. pestis* CO92 (**Table 1**). However, as observed for the initial strains we tested, conjugation to AuNPs decreased the MIC of Kan against both strains, reducing the MICs by 13.50- and 42.40-fold for *Y. pestis*::Km and *P. aeruginosa* UNC-D, respectively. Importantly, in all cases conjugation to AuNPs improved the MIC of Kan to within a range for aminoglycosides that is considered sensitive by the CLSI guidelines (“M100-S25: Performance Standards for Antimicrobial Susceptibility Testing; Twenty-Fifth Informational Supplement” National Committee for Clinical Laboratory Standards, 2015).

**Table 1:** Efficacy of Kan-AuNPs against bacterial strains [μg/ml].

Bacterial Strain	Kan MIC	Kan-AuNPs MIC^a^	Fold change
*S. bovis*	512	9.8	52.2
*S. epidermidis*	64	6.3	10.2
*E. aerogenes*	64	5.6	11.4
*P. aeruginosa* PA01	50	6.7	7.5
*P. aeruginosa* UNC-D	140	3.3	42.4
*Y. pestis* CO92	5	1.7	2.9
*Y. pestis* CO92::Km	180	13.3	13.5


*^a^Minimum inhibitory concentration (MIC) represents the concentration of Kan present in the nanoparticles.*

**FIGURE 2:**
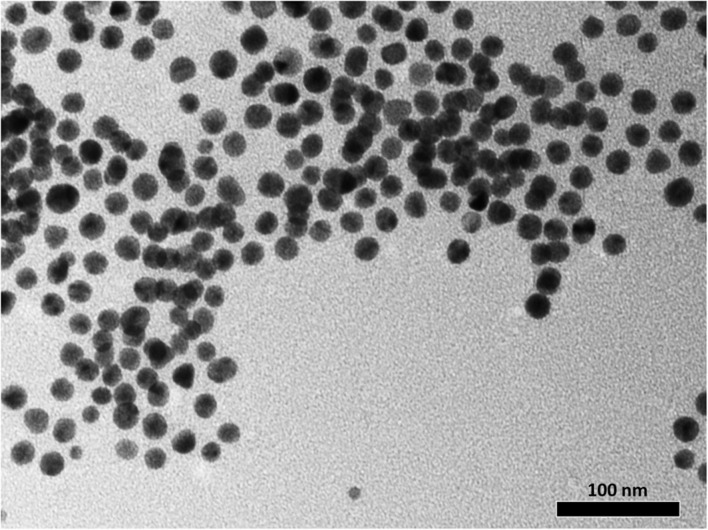
**Illustration of the morphological characterization of synthesized citrate-AuNPs.** The TEM micrograph shows the formation of well-dispersed spherical citrate-AuNPs in the size range of 20 ± 5 nm. A core size that is quite comparable to that of the Kan-AuNPs.

In addition to drug delivery capabilities, several studies have suggested that AuNPs have inherent antimicrobial activities (Zhou et al., 2012; Lima et al., 2013; Bindhu and Umadevi, 2014; Ortiz-Benitez et al., 2015) which may have contributed to the decreased MIC of the Kan-AuNPs. To determine the antimicrobial potential of AuNPs, we synthesized citrate-AuNPs of similar size to the Kan-AuNPs (20 ± 5 nm) (**Figure 2**). MICs for the citrate-AuNPs were determined against *S. epidermidis, S. bovis* and *E. aerogenes*. Unlike Kan-AuNPs, citrate-AuNPs were not able to inhibit the growth of any of these strains (MICs were >600 μg mL^-1^, which is at the dispersity limit of the citrate-AuNPs) (**Table 2**). The surface zeta potential of the citrate-AuNPs was determined to be -33.5 mV. While this indicates a highly stable nanoparticle, the fact that the electrostatic charge is negative may explain the high MIC as the negative surface charge of the nanoparticle repels the negatively charged surface of the bacterial membrane (Dickson and Koohmaraie, 1989; Silhavy et al., 2010). Together these data demonstrate that conjugation of Kan to AuNPs significantly decreases the MIC of the antibiotic and that the increased efficacy is independent from potential antimicrobial characteristics of the AuNPs.

**Table 2:** Efficacy of citrate-AuNPs compared to Kan-AuNPs [μg/ml^a^].

Bacterial strain	Citrate-AuNP MIC	Kan-AuNPs MIC	Fold change
*S. bovis*	>600	18.2	>33
*S. epidermidis*	>600	11.7	>51.3
*E. aerogenes*	>600	10.4	>57.7


*^a^Minimum inhibitory concentration (MIC) represents the concentration of Au present in the nanoparticles.*

**FIGURE 3:**
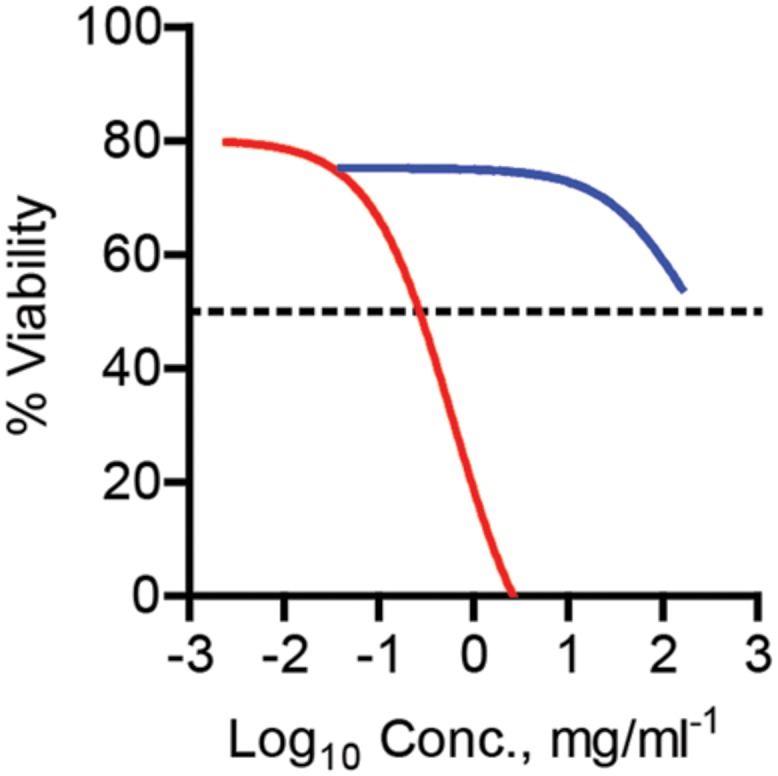
**Cytotoxicity of Kan-AuNPs.** To measure the potential cytotoxicity of Kan-AuNPs, Vero 76 cells were treated with increasing concentrations of Kan-AuNPs (red) or Kan (blue) (*n* = 3). Twenty four hours after addition of drug cell viability was determined using Alamar blue.

An effective antimicrobial needs to be inhibit bacterial growth at a concentration lower than the tolerated dose of the drug. One measure of tolerance is to determine the cytotoxicity of the drug in tissue culture. Toward this end, Vero 76 cells were treated with increasing concentrations of Kan-AuNPs and cell viability was determined 24 h after treatment. The concentration that resulted in a 50% decrease in viability (CC_50_) was calculated to be 609.6 μg/ml. The *in vitro* CC_50_ of the Kan-AuNPs is lower than Kan alone (**Figure 3**) but is >30-fold higher than the average MIC of Kan-AuNPs against the bacteria in (**Table 1**). The significantly lower MIC of Kan-AuNPs compared to the CC_50_ suggests that the effective dose will be lower than the minimum tolerated dose of the Kan-AuNPs *in vivo*, and therefore, suitable for future preclinical studies, especially against drug resistant bacteria that Kan alone is not efficacious.

### Kan-AuNPs Gain Entry into the Bacterial Cytosol

**FIGURE 4:**
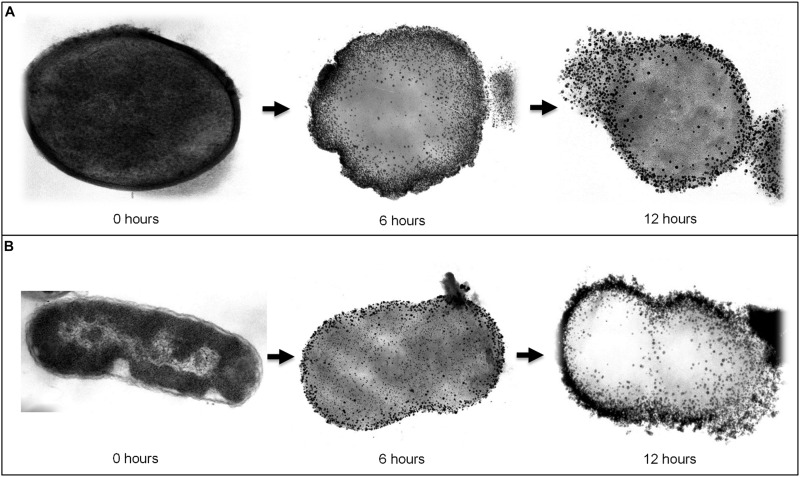
**Transmission electron microscope (TEM) images which visualize the morphological changes in bacteria upon treating with Kan-AuNPs at different intervals of time.** **(A)** Represents sequential images (from left to right) of Gram-positive, *S. epidermidis* bacteria treated with Kan-AuNPs (18.00 μg mL^-1^) after 0, 6, and 12 h of incubation. **(B)** Represents sequential images (from left to right) of Gram-negative, *E. aerogenes* bacteria treated with Kan-AuNPs (16.00 μg mL^-1^) after 0, 6, and 12 h of incubation. After 6 h of exposure, Kan-AuNPs were found to adhere and penetrate the bacterial cell wall which resulted in disruption of cellular environment leading to lysis of cell due to leakage of cellular components as observed after 12 h of exposure. The results were similar for both Gram-positive and Gram-negative bacteria.

**FIGURE 5:**
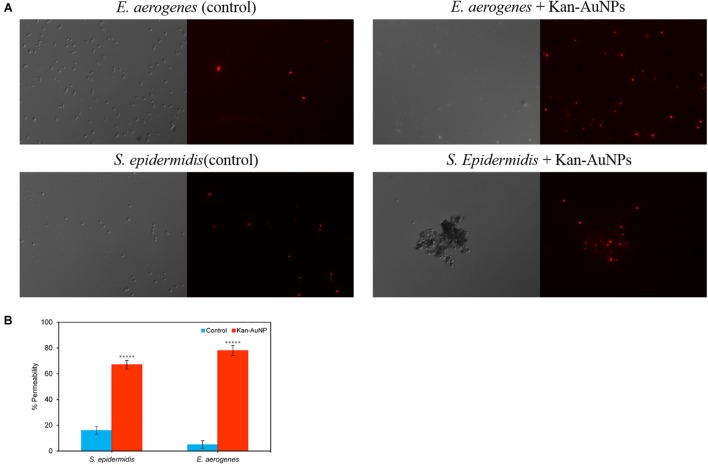
**Fluorescence images of Kan-AuNP induced cell membrane permeability using propidium iodide (PI) dye.** Upper panel represents Gram-positive bacteria, *S. epidermidis*, and lower panel represents Gram-negative bacteria, *E. aerogenes*. **(A)** For each image, the left half represents differential interference contrast mode, while the right half represents the corresponding fluorescence image. Untreated samples of respective bacteria with Kan-AuNPs were taken as control. **(B)** Represents a plot showing percentage permeability of *S. epidermidis* and *E. aerogenes* bacterial cells in presence and absence (Control) of Kan-AuNPs. ^∗∗∗∗∗^*p* ≤ 0.00001 (Student’s *t*-test, two tailed assuming unequal variances).

Assuming AuNPs promote delivery of Kan to bacterial cells, we would expect that Kan-AuNPs will associate and accumulate with the bacteria during treatment. Furthermore, the positive zeta potential of the Kan-AuNPs suggest that they may interact with negatively charged membrane of bacteria (Torchilin, 2014; Arakha et al., 2015). To track the localization of Kan-AuNPs, a representative Gram-positive and Gram-negative bacterium, *S. epidermidis* and *E. aerogenes*, respectively, were incubated with Kan-GNPs and samples were harvested at 0, 6, and 12 h post-treatment and imaged by transmission electron microscopy (TEM). For both bacteria, electron dense AuNPs were observed to accumulate at the membrane and within the cytosol (**Figure 4**). Furthermore, TEM images of later time points indicated compromised bacterial membrane integrity. To confirm loss of membrane integrity, Kan-AuNP-treated bacteria were incubated with propidium iodide (PI), a fluorescent dye that intercalates into the DNA of bacteria, but is excluded from bacteria with intact membranes. Fluorescence microscopy showed that 75 ± 10% of bacteria co-localized with PI after incubation with Kan-AuNPs, which was significantly higher than bacteria incubated with the equivalent MIC dose of Kan alone (**Figure 5**; *p* ≤ 0.00001). Together these data demonstrate that Kan-AuNPs accumulate within the bacterial membrane and cytoplasm, indicating that Kan-AuNPs are effectively delivered to the bacterium and to a location where Kan can interact with the protein synthesis machinery to directly inhibit bacterial growth.

## Conclusion

The increase in community and nosocomial acquired infections with drug resistant bacteria is a growing problem throughout the world. While continued support for efforts to identify novel antimicrobials is essential to combat these infections, technologies to improve the efficacy of existing antibiotics is also an important strategy to compliment the long and expensive process of *de novo* drug discovery. Here, we demonstrate for the first time an environmental friendly AuNP synthesis approach to quickly and easily conjugate antibiotics to AuNPs. The benefits of this approach include a synthesis methodology absent of the use toxic organic solvent and a single-step methodology. Furthermore, because of its simplicity, this synthesis approach is amenable to Good Manufacturing Practices (GMP) and easily scalable. Finally, as this process can use a wide variety of functional groups to mediate the reducing and capping steps in the synthesis, it is also applicable to the conjugation of many different classes of antibiotics. Thus, we postulate that this strategy will allow us to improve the efficacy of multiple antibiotics against drug resistant bacteria.

Using Kan as a proof of concept for this approach, we demonstrated that AuNP conjugation dramatically improved the efficacy of the antibiotic against both Kan sensitive and Kan resistant bacteria. Furthermore, we showed broad spectrum improvement that impacted both Gram-negative and Gram-positive bacteria. Importantly, the MICs that we observed were below the CLSI breakpoint for Kan (“M100-S25: Performance Standards for Antimicrobial Susceptibility Testing; Twenty-Fifth Informational Supplement” National Committee for Clinical Laboratory Standards, 2015) and the CC_50_, strongly indicating that Kan-AuNPs will be efficacious *in vivo*. The mechanism(s) responsible for the increased activity is currently unknown. However, accumulation of the Kan-AuNPs in the bacterial membrane and within the bacterial cytosol indicates that the AuNPs may facility entry across the bacterial membrane or increase the local concentration of the antibiotic compared to antibiotic alone. This local increase in concentration may be enough to overcome resistance mechanisms. Alternatively, in many bacteria, highlighted here by *P. aeruginosa*, drug efflux pumps are a major mechanism of antibiotic resistance. It is possible that conjugation to AuNPs inhibits the ability of these systems to efflux the antibiotic, allowing the antibiotic to remain within the bacteria in spite of these pumps. Finally, it is possible that inhibition is a combination of antibiotic activity and cell membrane disruption by the AuNPs. We are currently in the process of better defining the mechanism(s) of increased Kan efficacy. However, and importantly, our data indicate that the conjugation process does not inactivate the Kan, as citrate-AuNPs of similar size do not have comparable antimicrobial activity as Kan-AuNPs. Therefore, regardless of the mechanism, it appears to be dependent on conjugation to an active antibiotic.

## Author Contributions

JP, HW, MC, WH, ST, HM, FC, and VB performed experiments. JP, RD, and ML designed experiments, analyzed data and made conclusions. JP, ML, and RD drafted the manuscript.

## Conflict of Interest Statement

The authors declare that the research was conducted in the absence of any commercial or financial relationships that could be construed as a potential conflict of interest.
